# Editorial: Interplay between the heart and the immune system: Focus on heart rhythm regulation

**DOI:** 10.3389/fphys.2022.981499

**Published:** 2022-08-10

**Authors:** Lubov Mitrofanova, Sergey Popov

**Affiliations:** ^1^ Almazov National Medical Research Centre, Saint Petersburg, Russia; ^2^ Cardiology Research Institute, Tomsk National Research Medical Center, Russian Academy of Sciences, Tomsk, Russia

**Keywords:** atrial fibrillation, inflammation, pathogenesis, macrophages, artificial intelligence

Activation or dysregulation of the immune system plays an important role in the development and progression of many cardiovascular diseases and cardiac arrhythmias. Atrial fibrillation (AF) is the most common cardiac arrhythmia. Inflammation contributes to both electrical and structural atrial remodeling and thrombosis in patients with AF, and therapy targeting specific inflammatory cascades may be a potential strategy to prevent and treat AF (Hu et al., 2015). However, AF has a complex and multifactorial developmental mechanism that involves more than just inflammation.

Clinical studies have shown that AF can be triggered by autonomic stimulation, bradycardia, atrial premature beats, tachycardia, accessory pathways, and acute atrial stretch (Weil and Ozcan, 2015). The electrical or focal mechanisms of AF include abnormal automatism, trigger activity and multiple variable reciprocal patterns (Wakili et al., 2011; January et al., 2014). Reduced L-type Ca^2+^ (ICaL) current, Ca^2+^ overload, changes in K^+^ current (IKACh, IK1), Na^+^ current (INa), and transient outward current (Ito) have each been reported in AF (Nattel, 2002). In turn, ionic currents can directly affect the function of cardiomyocyte mitochondria, since it was previously shown that a high content of Ca^2+^ inhibits mitochondrial respiration, dissipates the membrane potential, and suppresses ATP production (Holmuhamedov et al., 2001; Jahangir et al., 2001). Remodeling of the calcium cycle can contribute to the progression of AF to persistent and activate profibrotic pathways (Denham et al., 2018).

In addition to the electrophysiological aspects, the complex anatomical structure of the atria is of great importance (Platonov et al., 2008). It is known that the conduction of an electrical impulse goes through wide muscle bundles with a parallel arrangement of muscle fibers (Anderson et al., 1983). The anatomy of the pulmonary veins promotes re-entry due to the combination of abrupt changes in fiber orientation and reduced electrical connectivity between muscle bundles creating areas of heterogeneous conduction velocity and localized block (Hocini et al., 2002; Arora et al., 2003; Hsueh et al., 2013).

Mounting evidence has shown that cardiomyocytes are not the only cells of the heart with an electrical activity. Recent studies have shown that fibroblasts express voltage-gated sodium channels that provide inward current flow (INa) (Chatelier et al., 2012; Koivumäki et al., 2014; Sánchez et al., 2019). Although they are not electrically excitable, they can affect the electrophysiological properties of myocytes (Gaudesius et al., 2003). Fibroblasts have been found to electrically react with myocytes through gap junctions (Jousset et al., 2016). Fibroblasts become active under inflammatory response and then differentiate into myofibroblasts (Chacar et al., 2017) by different pathways (Jalife and Kaur, 2015). Myofibroblasts differ from fibroblasts in that they develop contractile proteins and exhibit a more depolarized resting membrane potential (Salvarani et al., 2017) and greater membrane capacitance (Sridhar et al., 2017).

Recent investigations have shown that the myocardium contains a 3D network of telocytes that tightly cover cardiomyocytes and are involved in contacts with all types of cells and structures (Hinescu and Popescu, 2005; Popescu et al., 2005; Cretoiu et al., 2014). In addition to gap junctions, telocytes have point, nanocontacts, and planar contacts with cells. They can penetrate the basement membrane that envelopes two P cells of the cardiac conduction system together (Mitrofanova et al., 2018). Sheng et al. (2014) demonstrated that atrial and ventricular telocytes express Ca^2^⁺ activated high conductive K⁺ current (BK(Ca)) and internal rectifying K⁺ current (IK(ir)), but not transient external K⁺ current (I (to)) and an ATP-sensitive potassium current (K (ATP)). The results of these authors showed that functionally competent K^+^ channels are present in human heart telocytes, and their modulation may be of significant importance in myocardial physiopathology.

Cardiac macrophages have been recently demonstrated to also play an important role in both electrophysiology and arrhythmogenesis. Cardiac macrophages are a heterogeneous group of immune cells including resident macrophages derived from embryonic and fetal progenitors and recruited macrophages derived from circulating bone marrow monocytes (Xia R. et al.).

Several studies have demonstrated specific macrophage-dependent mechanisms regulating electrical, structural, or autonomic remodeling leading to arrhythmias (Monnerat et al., 2016; Liu et al., 2019; Miyosawa et al., 2020; Hiram et al., 2021). Hulsmans et al. (2017) have shown that cardiac macrophages facilitate electrical conduction through the distal atrioventricular node, where conducting cells densely intersperse with elongated macrophages expressing connexin 43. Disruption of the macrophage-cardiomyocyte interaction results in impaired electrical conduction in the AV node. Could it be telocytes? Simon-Chica et al. (2022) revealed that resident murine cardiac macrophages express potassium channels including Kv1.3, Kv1.5, and Kir2.1 which establish several inward and outward rectifying currents. The interaction between macrophages and cardiac fibroblasts regulates the balance of cardiac fibrosis (Van Linthout et al., 2014).

Currently, many researchers believe that inflammatory processes are key factors in the pathophysiology of AF [Ihara and Sasano]. The incidence of AF increases in the presence of systemic inflammation (Christian et al., 2008). Cases of occurrence of AF in COVID-19 are described (Gaine et al., 2021). AF may be associated with chronic inflammation (Choi et al., 2019; Lazzerini et al., 2019). Inflammation can be systemic or focal (eg, myocarditis) (Subahi et al., 2019). It is quite expected, that increased levels of inflammatory markers (e.g., CRP, IL-6, IL-8, TNF-α etc.) are described in patients with AF (Patel et al., 2010).

Traditional clinical practice has failed in several areas such as identifying patients at risk for AF or patients with concomitant undiagnosed paroxysmal AF. New approaches using artificial intelligence may provide new tools to solve some of these old problems (Isaksen et al., 2022). In this regard, the study of Jiang et al. is extremely relevant. The authors showed that aging, overweight, hypertension, diabetes and smoking seemed to be associated with high CRP levels. This study proved the existence of inflammation-related changes in cardiac electrophysiological signals.

The autonomic nervous system plays an important role in the onset and maintenance of AF. Catheter ablation of ganglionated plexi (GP) in the left and right atrium has been proposed in varied clinical conditions. The benefit of adding autonomic ganglion ablation to the standard pulmonary vein exposure procedure for patients with paroxysmal AF is supported by both experimental and clinical data (Stavrakis et al., 2015; Rebecchi et al., 2021). The original experimental study of Ma et al. convincingly demonstrates that local administration of TRAM-34, an inhibitor of intermediate-conductance KCa channels (SK4), into the atrial GP can suppress GP activity and AF vulnerability during rapid atrial pacing. The effects of TRAM-34 may be related to the macrophage polarization and inflammatory response of GP. This study once again underlines the multilevel and complexity of the pathogenesis of AF and opens up new prospects for treatment.

In sum, the articles in this Research Topic demonstrate the complexity of the pathogenesis of AF, which has many levels: electrophysiological, anatomical, histological, cellular, ultrastructural and molecular. Inflammation is the key link. Further study of the pathogenesis of cardiac arrhythmias with the involvement of genetics, the study of the role of transcription factors, LncRNAs and others will allow moving from surgery and pharmacology to gene therapy.
